# Acupuncture modulates the gut microbiota in Alzheimer’s disease: current evidence, challenges, and future opportunities

**DOI:** 10.3389/fnins.2024.1334735

**Published:** 2024-03-01

**Authors:** Long Yan, Hong Li, Yulin Qian, Qidi Liu, Shan Cong, Baomin Dou, Yu Wang, Meng Wang, Tao Yu

**Affiliations:** ^1^The First Teaching Hospital of Tianjin University of Traditional Chinese Medicine, Tianjin, China; ^2^National Clinical Research Center for Acupuncture and Moxibustion, Tianjin, China; ^3^Tianjin University of Traditional Chinese Medicine, Tianjin, China

**Keywords:** acupuncture, Alzheimer’s disease, gut microbiota, probiotics, inflammation

## Abstract

Alzheimer’s disease, one of the most severe and common neurodegenerative diseases, has no effective cure. Therefore it is crucial to explore novel and effective therapeutic targets. The gut microbiota - brain axis has been found to play a role in Alzheimer’s disease by regulating the neuro-immune and endocrine systems. At the same time, acupuncture can modulate the gut microbiota and may impact the course of Alzheimer’s disease. In this Review, we discuss recent studies on the role of acupuncture on the gut microbiota as well current challenges and future opportunities of acupuncture as potential treatment for the prevention and treatment of Alzheimer’s disease.

## 1 Introduction

Alzheimer’s disease (AD) is the leading cause of dementia in the elderly. As populations continue to age worldwide, AD has rapidly become one of the most costly diseases to health systems and carries very high emotional burden to families and caregivers (Scheltens et al., 2021). About 47 million people worldwide are currently estimated to suffer from dementia, a figure that is expected to triple by 2050 (Arvanitakis et al., 2019; Ashrafian et al., 2021). AD is a neurodegenerative and neuroinflammatory disease of the central nervous system characterized by extracellular amyloid β (Aβ) plaques and hyperphosphorylated microtubule-associated protein tau (Ferrari and Sorbi, 2021). The primary clinical manifestation of AD is a decline in cognitive-behavioral abilities, leading to loss of independence in life; however, there is no accepted view of the underlying pathology (Armstrong, 2019). AD is considered a multifactorial disease, with risk factors that include age, genetic factors, head injury, infections, environmental factors (such as heavy metals and exposure to other polluters), and psychological disorders (Heneka et al., 2015; Scheltens et al., 2021). Epidemiological data shows that the onset of AD is most common in people older than 65 years, suggesting that age is the strongest risk factor for developing AD (Guerreiro and Bras, 2015; Khanahmadi et al., 2015). The presence of Aβ plaques, which are thought to be critical to the development of AD, in different areas of the brain triggers synaptic deficits and neurodegeneration by activating autoimmune responses, ultimately leading to cognitive impairment (Khan et al., 2020). NFT is an abnormal filament of hyperphosphorylated tau protein that twists around each other to form paired helices (Brion, 1998), causing loss of function of the tau protein and negatively affecting axonal function (Utton et al., 2005), leading to Aβ accumulation and neurodegeneration. Central neuroinflammation is characterized by activation astrocytes and microglia, with the pro-inflammatory effect of M1 microglia playing a particularly important role (Habib et al., 2020; Merighi et al., 2022). Indeed, microglia are now one of the emerging therapeutic targets for AD (Mo et al., 2021).

Research in recent years has revealed a correlation between gut microbiota and the development of AD. This is currently one of the critical research areas in the field and a promising therapeutic target (Kim et al., 2020). Some evidence has demonstrated that gut microbiota is associated with the pathophysiology of neurodegenerative lesions (Kowalski and Mulak, 2019; Megur et al., 2020; Doifode et al., 2021). However, the specific mechanism of action underlying this association is unclear. Acupuncture is a traditional therapy in Chinese medicine with a long history of improving dementia (Yan et al., 2022). Acupuncture has been shown to improve cognition in patients with AD as well as improving their ability to live independently with effects that are similar to those obtained with drugs (Huang J. et al., 2020; Wang L. Y. et al., 2020; Wang Y. Y. et al., 2020). Studies have also shown that acupuncture reduces Aβ deposition, improves cholinergic neurotransmission, and stimulates activation of cognitively relevant brain regions to ameliorate AD (Cai et al., 2019; Tu et al., 2019; Wang Y. et al., 2020).

This paper reviews recent evidence on the role of acupuncture to modulate gut microbiota as a therapy for AD. We discuss the effects of acupuncture and the gut microbiota on neuromodulatory mechanisms and immune regulation in AD, and suggest that acupuncture combined with modulation of the gut microbiota may be beneficial in the treatment of AD in the future.

**Figure 1:**
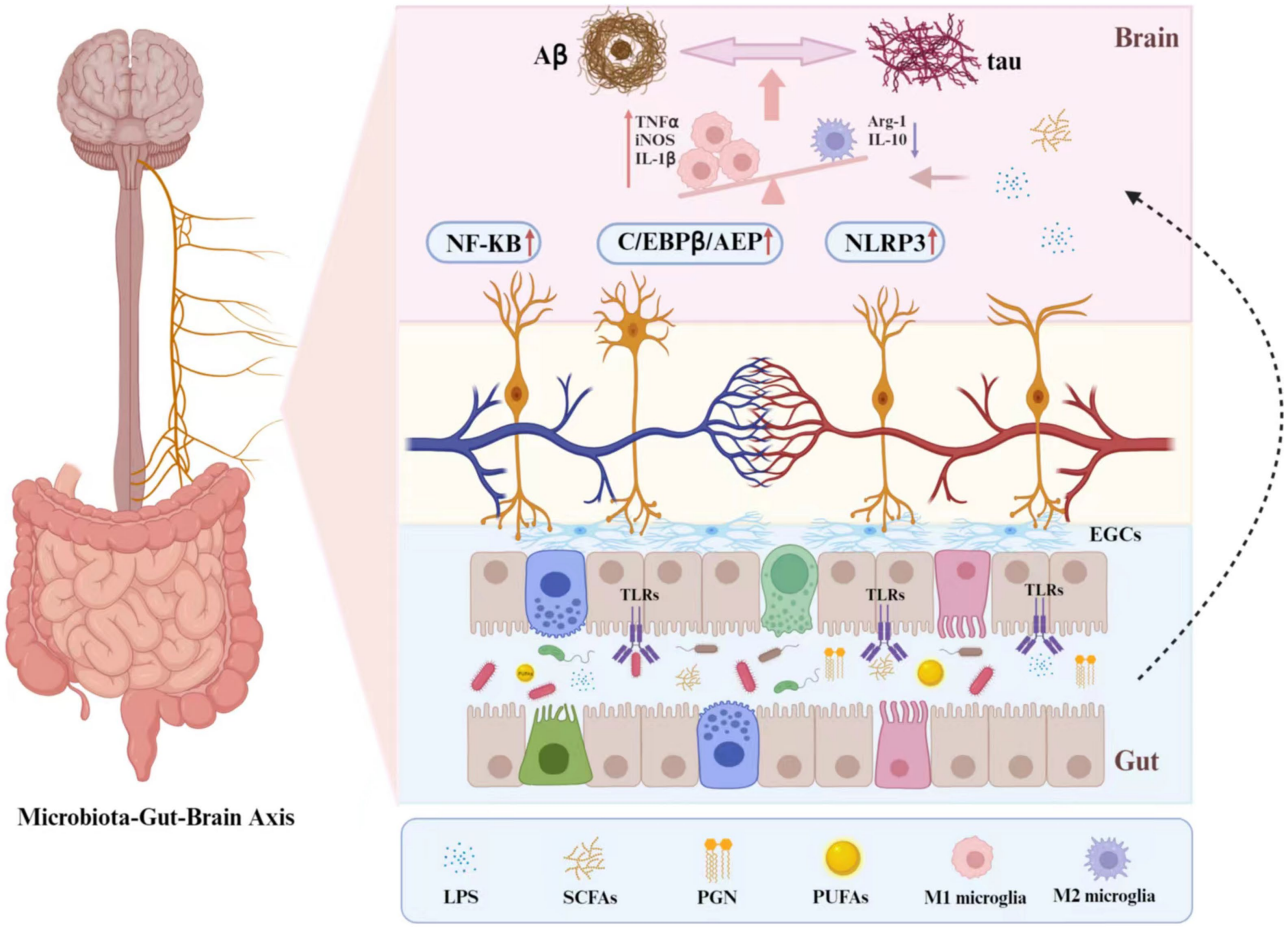
The relationship between Alzheimer’s disease and the gut microbiota. The brain-gut axis is a two-way communication system between the brain and the gut. The vagus nerve acts as a communication hub to establish a connection between the brain and the gut. The EGCs and TLRs in the intestinal epithelium can work with the vagal nerves to detect changes in gut signals and then complete the transmission of information from the brain to the gut. TLR and PGN in the intestinal environment can act as receptors of the microbial immune response and induce immune responses in various organs, including the brain, via activation of local immunity. Additionally, during AD pathology, gut microbes produce derived products such as SCFAs, LPS and PUFAs, which can permeate the intestinal epithelium and the blood–brain barrier and enter the brain via systemic circulation. This process can influence the polarization of microglia toward a pro-inflammatory direction, activate inflammatory pathways in the brain and upregulate the levels of inflammatory factors, leading to the deposition of pathological products such as A β in the brain. Simultaneously, the heightened inflammatory response in the brain also impacts the gut microbiota, creating a two-way regulation that ultimately triggers AD.

Figure 1 shows the relationship between Alzheimer’s disease and the gut microbiota.

## 2 Acupuncture modulates the gut microbiota in Alzheimer’s disease

### 2.1 Effect of acupuncture on Alzheimer’s disease

Acupuncture is now widely used in the treatment of cognitive disorders such as Alzheimer’s disease and general mild cognitive impairment (Du et al., 2020; He et al., 2021; Wang X. S. et al., 2021). Acupuncture is an extremely important complementary therapy in China. Acupuncture as a treatment for AD is documented in “*Zhenjiu Jiayi Jing*” by Huangfu Mi of the Western Jin Dynasty of China and “*Zhenjiu Dacheng*” by Yang Jizhou in the Ming Dynasty. “*Zhenjiu Jiayi Jing*” is the earliest writing on acupuncture in Chinese history, which records that acupuncture was used in the treatment of Alzheimer’s disease as early as the Western Jin Dynasty (Changlin et al., 2023). The pathway of the Du (GV) and bladder (BL) meridians passes through the brain and is currently seen as the most important meridian for the treatment of AD (WuLi et al., 2021). With the development of modern Chinese medicine, some well-known theories have gradually emerged in the treatment of AD. “Dongdu Qishen” acupuncture is one of the more recognized acupuncture theories, which mainly stimulates GV meridian points, such as GV20, GV29, GV26, and so on (Cai et al., 2019). Another acupuncture theory for the treatment of AD is Sanjiao acupuncture, proposed by Professor Han Jingxian, which is based on “trifocal dysfunction - aging,” with CV17, CV12, BL24, SJ 5, SP10, ST36 as the main stimulation points; these points can affect synaptic plasticity and neuroinflammation to improve learning and memory function (Yu W. et al., 2021).

Studies have shown that acupuncture can prevent and alleviate Alzheimer’s disease to some extent (Yu C. C. et al., 2021). The clinical study in people using fMRI, acupuncture LR3 and LI4 were found to trigger low-frequency amplitude changes in brain regions associated with cognition in AD patients and enhance the functional link between the hippocampus and the anterior central gyrus (Zheng et al., 2018).

**Figure 2:**
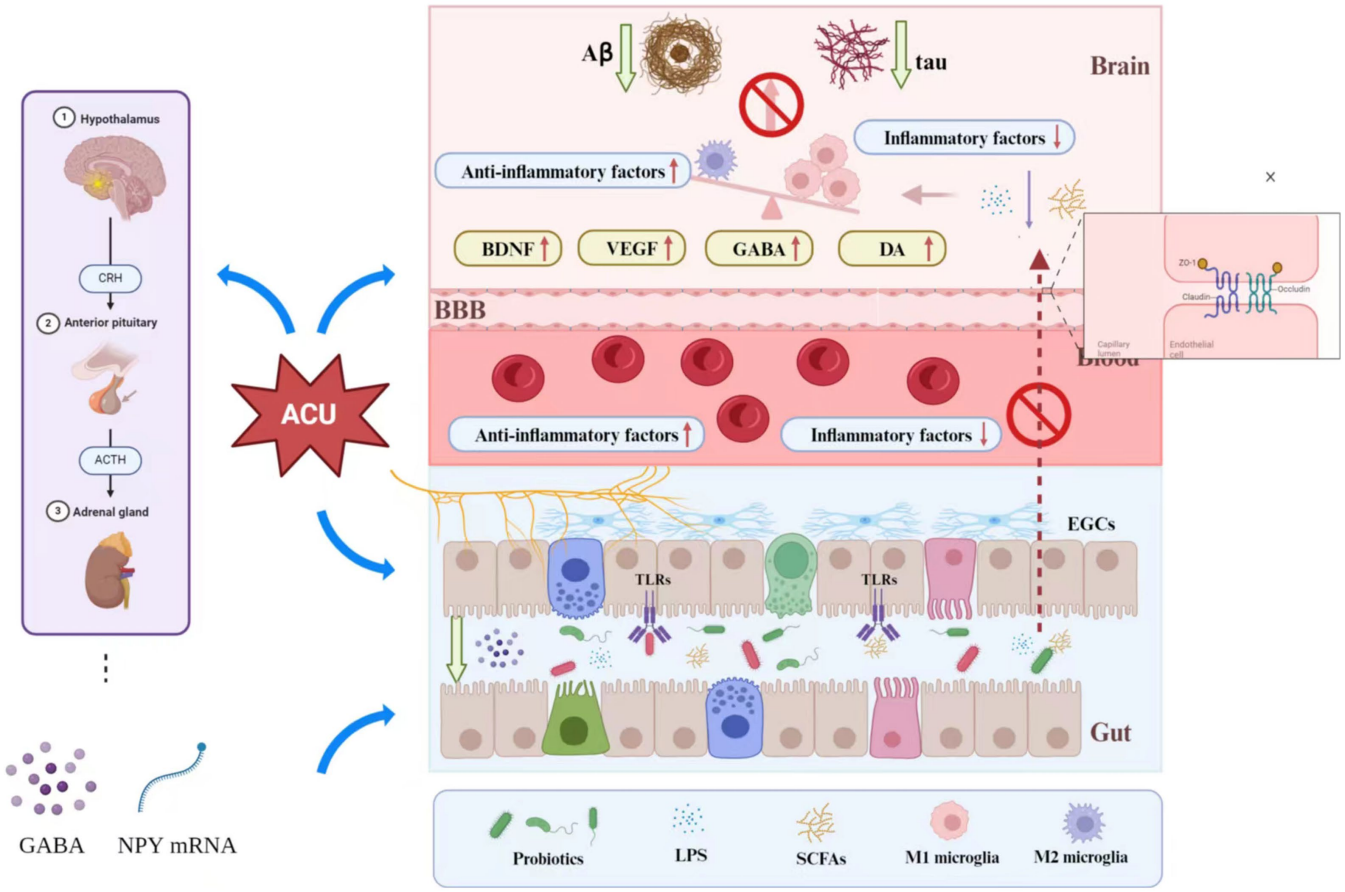
Mechanism of acupuncture in the treatment of Alzheimer’s disease. Acupuncture can affect the messages via the BGM axis to influence AD in the following several ways: (1) Acupuncture can directly (or through the vagus nerve) affect the structure and function of gut microbiota to further reduce intestinal permeability, and may also affect the inflammatory response of the CNS and Aβ expression through this process. (2) Acupuncture can modulate the activation of the HPA axis, which further affects the expression of γ-aminobutyric acid and NYP mRNA to reduce intestinal permeability. (3) Acupuncture can increase the expression of tight-binding proteins in the BBB (such as the expression of ZO-1 and occlusion proteins) to reduce BBB permeability. (4) By modulating the levels of probiotics, acupuncture can affect inflammation in the CNS. The probiotics also can reduce LPS secretion to reduce intestinal permeability. (5) Acupuncture can upregulate BDNF, VE GF, GABA and DA levels to influence neural functional remodeling.

Preclinical studies shows that acupuncture can also change the ultrastructure of hippocampal dentate neurons and astrocytes in AD mice, alleviating mitochondrial swelling and endoplasmic reticulum dilation (Tang et al., 2019). The transition of microglial phenotype is important for the prevention and treatment of AD, and acupuncture has been shown to regulate this process, allowing differentiation from anti-inflammatory M2 to improve cognitive and recognition functions in AD rats (Xie et al., 2021). Activation of pro-inflammatory microglia in AD leads to decreased synaptic plasticity and synaptic loss in nerve centers (Jones and Lynch, 2015), and electroacupuncture (EA) intervention in bilateral KI3 of 5XFAD mice can upregulate synaptic PSD-95 protein expression and inhibit degradation of synaptic ultrastructure, thereby improving synaptic plasticity and cognitive function (Cai et al., 2019). The transcription factor EB (TFEB) is a key regulator of the autophagy-lysosomal pathway (ALP) (Settembre et al., 2011), which is linked to development of AD (Boland et al., 2018). EA located in GB13 and GV24 can improve memory in 5XFAD mice, via inhibition of the activation of MTOR (mammalian target of rapamycin) kinase complex 1 (MTORC1), MAPK1/ERK2 (mitogen-activated protein kinase 1), AKT (AKT serine/threonine kinase 1), increasing expression of ALP and reducing Aβ deposition and neuronal apoptosis in the hippocampus (Zheng et al., 2021). Based on these results, it can be hypothesized that acupuncture inhibits neuroinflammatory lesions and improves cognitive dysfunction in AD via different mechanisms such as anti-inflammatory, mechanisms, enhancement of neuroplasticity, and improvement of microstructure of brain regions related to cognition. Figure 2 depicts the mechanism of acupuncture in the treatment of Alzheimer’s disease.

### 2.2 Acupuncture modulates the gut microbiota in Alzheimer’s disease

The term “gut microbiota” refers to the symbiotic microbial community that colonizes the gastrointestinal tract consisting of bacteria, fungi, archaea, viruses, and protozoa that are symbiotic with our intestinal tract (Varesi et al., 2022). With a mass of 1–2 kg, comparable to that of the human brain, the gut is the most significant bacterial reservoir in the body (Stilling et al., 2014).

Clinical studies have found the relationship between AD and the gut microbiota. Some studies have found that the gut microbial community differs between AD patients and healthy people (Liu et al., 2020; Jeong et al., 2022). People with AD have been shown to have significantly reduced diversity (Vogt et al., 2017; Kim et al., 2022). A study (Ling et al., 2020) investigating the structure–function of microorganisms in 100 AD patients and 71 normal people found that the bacterial diversity and composition of fecal microorganisms were also significantly reduced in AD patients. Some intestinal microorganisms (such as the *phylum Aspergillus* and its genera *Gamma Aspergillus* and *Enterobacteriaceae*) were also negatively correlated with MMSE and MoCA scores, raising the question on whether the structure of the intestinal microbiota is directly related to the decline in cognitive performance and actively participates in the development and progression of AD (Vogt et al., 2017; Liu et al., 2019).

In addition, Preclinical studies have also found interesting results. Genetic sequencing studies have revealed significant differences in ileal and colonic alpha diversity, beta diversity, and dominant microbial diversity between AD rats and normal rats (Xu et al., 2022) and showed correlation between the development and progression of AD and dysregulation of the gut microbiota (Favero et al., 2022). It has been proposed that the pathological processes associated with cognitive deficits in AD may be related to the dysregulation of pyrimidine metabolism in the gut microbiota (Feng et al., 2022). Metabolites produced by intestinal flora, such as trimethylamine-nitrogen-oxide (TMAO), have been shown to increase beta enzyme activity and up-regulate Aβ secretion, further exacerbating the development of AD (Gao et al., 2019). Oral administration of Korean red ginseng improved cognitive function in Tg2576 mice. These mice have also been reported to have altered gut microbiota diversity (Lee et al., 2022), suggesting a bidirectional mediating effect of the gut microbiota AD and leading to the hypothesis that correcting the gut microbiota structure may be essential for treating AD (Tables 1, 2).

**Table 1:** Relationship between gut microbiota and central nervous system alterations in AD patients.

Research	Microorganisms	Intervention targets	Impact
Jeong et al. (2022)	Phylum Pachychophyllum, Actinobacilli	AD patients	Correlation with AD score
Vogt et al. (2017)	Phylum Pachychophyllum, Bacteroides, Bifidobacterium	AD patients	Correlation with Aβ deposition, phosphorylated tau levels
Ling et al. (2020)	Clostridium prabio, Bifidobacterium	AD patients	Associated with the release of pro-inflammatory and anti-inflammatory factors
Liu et al. (2019)	Phylum Pachychophyllum, *Bacillus deformans*	AD patients	Impact AD-related scores

**Table 2:** Relationship between gut microbiota and central nervous system alterations in animal studies.

Research	Microorganisms	Intervention targets	Impact
Kim et al. (2022)	Bacteroides, *Lactobacillus*, *Clostridium perfringens*	AD rats	Associated with Aβ deposition
Bravo et al. (2011)	*Lactobacillus*	Mice	Increased GABA concentration, decreased corticosterone and depressive state in hippocampus
Wang S. et al. (2020)	*Lactobacillus* enterica, *Lactobacillus royi*	CSDS Mice	Affects plasma IL-6 and synaptophysin

Acupuncture can modulate the gut microbiota (Table 3). A clinical study of 30 patients with subjective cognitive decline (SCD), GV20, GV24, ST36, PC6, GB20, and RN12 showed that, the diversity of intestinal flora in the stool of patients in the observation group differed significantly before and after treatment with bifidobacteria, and bifidobacteria were associated with improvement in clinical cognitive scores (Wang T. et al., 2022).

**Table 3:** Research on the regulation of intestinal microbiota by acupuncture.

Study	Microorganism	Intervention targets	Impact
He et al. (2021)	*Lactobacillus*, Bifidobacterium, Bacteroides, *Escherichia coli*	AD mice	Associated with the release of pro-inflammatory and anti-inflammatory factors
Jiang et al. (2021)	Bacteroides, *Clostridium*, Proteus, Iron Bacteria	SAMP8 mice	Associated with the release of pro-inflammatory and anti-inflammatory factors
Zhang et al. (2022)	Bacteroides, Firmicutes, Proteobacteria, Campylobacter, Actinobacteria	APP/PS1 mice	Associated with the release of pro-inflammatory and anti-inflammatory factors
Yang et al. (2022)	Bacteroides, Firmicutes, Actinobacteria, Porphyromonadaceae, Helicobacteraceae, *Lactobacillus*	APP/PS1 mice	Associated with Aβ deposition, phosphorylated tua levels, pro-inflammatory and anti-inflammatory factors
Hao et al. (2022)	Bacteroidetes, Proteobacteria, Escherichia-Shigella	APP/PS1 mice	Associated with the expression of factors such as glial fibrillary acidic protein (GFAP), lipopolysaccharide (LPS) and TNF-α

Preclinical studies have found similar results. A basic study has also shown that electroacupuncture can increase the levels of *Lactobacillus* and thus enhance the gut microbiota alpha diversity index (Wei et al., 2019). Electroacupuncture has been shown to reduce intestinal epithelial cell (IEC) apoptosis and permeability by increasing intestinal microbiota diversity and restoring community structure (Wang L. et al., 2020). This study was a four-week intervention in APP/PS1 mice by needling GV20, LI4, BL13, BL20, BL23, ST36, and SP6. 16srDNA sequencing revealed alterations in Bacillus and thick-walled bacteria counts in APP/PS1 mice following needling, and enhanced spatial memory and affected Aβ and tau protein expression in APP/PS1 mice but also reduced serum LPS concentrations and IL-10 levels (Yang et al., 2022). Another study found that acupuncture improved the learning and memory abilities of APP/PS1 mice by up-regulating *Bacteroidota* and down-regulating *Proteobacteria* and *Firmicutes.* The study suggests that acupuncture may have a potential impact on the gut microbiota as a means of modulating cognitive functions (Zhang et al., 2022). Furthermore, the EA intervention was found to impact cognitive function by modifying the gut microbiota composition (specifically Lactobacillus and Bifidobacterium) in a rat model of AD. Additionally, the result showed that the EA reduced colonic 5-HT levels and increased hippocampal 5-HT levels. And the expression of hippocampal JNK pathway-related proteins was also significantly inhibited by EA (Xiao et al., 2023). Stimulation of GV30 and GV29 in SAMP8 mice for 15 consecutive days showed that that EA intervention modulated cognitive function in these mice, as seen by the Morris water maze assay and 16S rDNA sequencing. In this study, the ratio of *Clostridium perfringens* and *Clostridium perfringens* was altered and the levels of pro-inflammatory factors such as IL-1β, IL-6 and TNF-α in the hippocampus and serum were found to be decreased (Jiang et al., 2021). Another related study suggested that the mechanism by which EA modulates cognitive function via the microbiota-gut-brain axis pathway might be related to TLR4/NF-κB and suggested that GV20 and ST36 could enhance this effect in AD rats (He et al., 2021). Warm acupuncture, another branch of acupuncture therapy, can also improve bacterial diversity and gut composition (Yu et al., 2022). Altogether, results from basic research and clinical interventions suggest that acupuncture can modulate gut microbiota and improve cognitive function. Although the exact mechanism of action remains unknown, the brain-gut-microbiota axis appears to play an important role. Therefore, modulation of the gut microbiota via acupuncture could be a potential therapy strategy for AD.

### 2.3 Acupuncture modulates the vagus nerve to suppress the inflammatory response of the intestinal microbiota

An important study in the field indicated that ingestion of *Lactobacillus* strains modulates GABA(Aα2) mRNA expression in the prefrontal cortex, amygdala, and hippocampus in mice. In contrast, no neurochemical and behavioral effects were seen in subdiaphragmatic vagotomy (SDV) mice (Bravo et al., 2011). These results suggest that vagal afferent and efferent fibers are the primary information pathways between the gut microbiota and the brain (Margolis et al., 2021). The same team discovered that an enteric glial cell (EGC) called Neuropod cell can form synapses with the vagus nerve to connect to brain structures, through which the central nervous system can receive information from the gut (Kaelberer et al., 2018). Specifically, the EGC plays a critical role in detecting bacterial levels, products in the gastrointestinal tract and signals from the gut microbiota through Toll-like receptors (TLRs); these signals are then transmitted to the central nervous system via vagal afferent fibers to regulate gastrointestinal motility and secretion (Abreu et al., 2005; Bonaz et al., 2018). In addition, the vagus nerve can also directly sense microbial signals from the gut (e.g., *Lactobacillus*, SCFAs) and transmit signals to the upstream center (Bravo et al., 2011; Goswami et al., 2018; Wang S. et al., 2020). On the other hand, stimulation of the vagus nerve can inhibit the expression of M1 macrophages, a type of pro-inflammatory macrophage. Through this process, the vagus nerve can alter intestinal permeability and microbiota (Yuan and Taché, 2017), an may, therefore, have a key role in the association between the intestinal microbiota and AD.

Acupuncture has been shown to modulate vagal nerve activity (Liu et al., 2021). A recent study showed that electroacupuncture modulates the expression of N-methyl-D-aspartate receptors (NMDAR) in the dorsal motor nucleus of the vagus (DMV) (Wang et al., 2018). After electroacupuncture treatment, rats model of intestinal ischaemia-reperfusion injury showed a significant increase in DA levels, decreased levels of TNF-α, and decreased malondialdehyde (MDA) and myeloperoxidase (MPO) intestinal levels, and decreased permeability of the gastrointestinal tract; these effects were not observed in rats after SDV (Li Y. et al., 2021). It was also found that EA enhanced gastrointestinal motility by modulating the vagus nerve after long-term electroacupuncture stimulation of ST36 bilaterally in rats with constipation (Wang et al., 2019b). The results of these two studies suggest the possibility that the vagus nerve can mediate EA in regulating of gastrointestinal responses. Furthermore, EA has also been shown to exert anti-inflammatory effects through activation of α7nAChR to reduce pro-inflammatory cytokines in the animal models with inflammation. This effect was diminished in mice after SDV, demonstrating the mediating effect of the vagus nerve on EA in inflammation (Zhang L. et al., 2021; Zhang Z. et al., 2021). It is worth mentioning that EA can also inhibit intestinal inflammation via the same pathway that mediates JAK2/STAT3 signaling pathway via the vagus nerve in mice with postoperative ileus (Yang F. et al., 2021). In summary, acupuncture protects the intestinal epithelial tissue by reducing intestinal permeability through stimulation of the vagus nerve (Yuan and Taché, 2017), and regulates intestinal function through vagal-mediated anti-inflammatory mechanisms (Du et al., 2013).

### 2.4 Influence of the gut microbiota on the neuro-immune system

Numerous studies have shown that T cells, B cells, and macrophages are widely distributed in the gut (Ivanov et al., 2008; Yang and Cong, 2021; Ren et al., 2023). The gut microbiota can significantly influence the development and response of these immune cells and regulate the release of pro-inflammatory factors in the gut (Yoo and Mazmanian, 2017; Zhang et al., 2019). Alterations in gut microbiota may increase peripheral phenylalanine and isoleucine concentrations, leading to infiltration of various types of immune cells, including T cells, B cells, neutrophils, dendritic cells, and monocytes (Liu et al., 2020). In contrast, CD4+ T helper cells are closely associated with the activation of M1 microglial cells in the development of AD (Wang et al., 2019a). Activated microglia increase the transcription of the pro-inflammatory factors IL-1β, IL-6, and TNF-α (Cornejo and Von Bernhardi, 2016), and, in turn, M1 microglia leads to blood–brain barrier dysfunction and leakage (Yu C. C. et al., 2021; Mou et al., 2022), further contributing to the neuroinflammatory response. Bacterial peptidoglycan (PGN), Lipopolysaccharide (LPS), and Gram-negative bacteria are essential factors in transmitting immune information between the gut and the brain (Varesi et al., 2022). TLR and PGN in the intestinal environment can act as receptors of the microbial immune response and induce immune responses in various organs, including the brain, via activation of local immunity (Varesi et al., 2022; Weagley et al., 2022). Under pathological conditions, LPS can act on the TLR in the brain via the blood circulation to produce a neuroinflammatory response (El et al., 2015). Short-chain fatty acids (SCFAs), which are metabolites of bacterial dietary fiber fermentation, can influence microglia-gut microbiota interactions during AD development, according to a recent net meta-analysis (Wang Q. et al., 2021). SCFAs have also been shown to promote Aβ deposition by modulating the microglia phenotypes (Chen C. et al., 2022).

Additionally, transplantation of intestinal microbes from AD patients into APP/PS1 mice led to increased expression of NLRP3, increased inflammatory factors in peripheral blood, central neuroinflammation, and increased cognitive impairment due to microglia activation in the hippocampus in these mice (Shen et al., 2020). A related study found that polyunsaturated fatty acid (PUFA) metabolites secreted by the gut microbiota, caused a neuroinflammatory response and accelerated cognitive impairment in 3xTg mice through activation of the C/EBPβ/asparagine endopeptidase pathway (Chen C. et al., 2022). This phenomenon can also lead to an increase in the number of activated microglia and macrophages (Soriano et al., 2022). Altogether, these results suggest that gut microbes may be responsible for triggering the neuroinflammatory response in the development of AD.

Acupuncture regulates the immune system by modulating the levels of local macrophages to produce anti-inflammatory effects (Li N. et al., 2021). Acupuncture ST36 can inhibit pro-inflammatory M1 macrophages while promoting anti-inflammatory M2 macrophages to regulate macrophage polarization in inflammatory tissues and inhibit the expression of pro-inflammatory factors (e.g., TNF-α, IL-1β) (Yang N. N. et al., 2021). In contrast, acupuncture intervention with GV20 combined with GV26 suppresses the expression of IL-1, IL-6, and TNF-α through α7nAChR (Liu Y. et al., 2022). Electroacupuncture has been shown to increase probiotics, decrease the ratio of Th17 cells in CD4 cells and increase Treg cell expression (Wei et al., 2019). These effects ultimately lead to reduced LPS in circulation (Bai et al., 2021), which in turn inhibits TNF-α, IL-6, IL-1β, and inducible nitric oxide sythase (iNOS) and elevates IL-10 levels (Wang L. et al., 2020), thereby inhibiting intestinal inflammation (Ma et al., 2021). In addition, acupuncture reduced intestinal inflammation by alleviating the disruption of the intestinal mucosal barrier in APP/PS1 mice, thereby improving cognitive function, even with effects comparable to those of probiotics (Hao et al., 2022).

A study by Chen et al. (2021), GV20, GV29, ST25, ST37, ST36, SP6, and LR3 were selected as acupuncture interventions in 20 human. In this study, 16S rRNA sequencing of their stool samples revealed reduced in levels of SCFAs following the acupuncture intervention. As we mentioned in the previous section, SCFAs can induce deposition of Aβ in microglia, leading to AD development. Evidence from preclinical studies also supports the effects of acupuncture on the central nervous system. A study showed that electroacupuncture stimulation of ST36 and SP6 blocked the TLR4/NF-κB pathway and release of NLRP3 inflammatory vesicles which can down-regulat IL-1β vesicle levels and attenuating hippocampal inflammatory responses in depression rats (Zhou et al., 2022). Notably, EA was also found to reduce ERK/JNK/P38 phosphorylation and to increase the expression of PICK1 and TLR complexes in hippocampal microglia to prevent LPS-induced neuroinflammation in epsis-associated encephalopathy rats (Mo et al., 2021). TREM2 is a novel target of microglial activation as it is highly expressed on microglia (Srinivasan et al., 2016). EA intervention up-regulates hippocampal TREM2 expression and has an anti-inflammatory effect in AD (Li et al., 2020). During AD development, electroacupuncture inhibits glial cell activation while modulating microglia polarization toward the M2 phenotype and increasing the anti-inflammatory factors IL-4 and IL-10 (Xie et al., 2021), Reducing the expression of NLRP3, cysteine-1, and IL-1β in the hippocampus (He et al., 2021). In addition to electroacupuncture, traditional acupuncture interventions in a mouse model of traumatic brain injury resulted in inhibition of M1 polarization in microglia and decreased inflammation by modulation of the RhoA/ROCK2 signaling pathway (Zhu et al., 2020). Furthermore, acupuncture also induces the expression of α7nAChR and activation of the downstream JAK2/STAT3 signaling pathway, which improves cognition through the cholinergic anti-inflammatory pathway (Cao Y. et al., 2021).

### 2.5 Effect of acupuncture on the blood–brain barrier

Damage to the blood–brain barrier may be involved in the initiation and progression of central system disease induced by gut microbiota dysfunction (Maes et al., 2019). Interestingly, the number of bacteria in the brains of AD patients was found to be significantly higher (5-10x higher) than that of the general population, suggesting that brain aging may be influenced by a decline in function of the immune system (Emery et al., 2017). Extensive data suggest that the blood–brain barrier in the hippocampus of humans and rodents begins to break down in mid-life (Senatorov et al., 2019). Damage of the blood–brain barrier is a precursor to early cognitive impairment and occurs independently of Aβ and tau accumulation (Montagne et al., 2019). A study suggested that the neuroinflammation observed in SAMP8 mice may be due to cytokine leakage into the brain due to an aged blood–brain barrier, with gut ecological dysregulation producing higher TMAO, further leading to central inflammation and cognitive deficits (Yang et al., 2019; Lanz et al., 2022). Other studies reported the existence of Gram-negative bacterial LPS in the brains of AD patients (Zhan et al., 2018) and indicated that the level of LPS in the blood of AD patients was three times higher than that of controls (Zhang et al., 2009). These changes are likely due to alterations in permeability of the intestinal and blood–brain barriers with age, allowing intestinal microorganisms and pathogens to enter the bloodstream into the brain (Liu et al., 2019). These events lead to an increase in LPS produced by Gram-negative bacteria in the intestine and blood, triggering endotoxemia and further inducing an increase in amyloid in the intestine, which in turn also increases the permeability of the intestinal and blood–brain barriers (Kalyan et al., 2022). The repetitive occurrence of this process results in a continuous transfer of cytokines and inflammatory factors from the gastrointestinal tract to the rest of the body (Pellegrini et al., 2018). Among these, LPS entering the blood and the brain can bind to microglia-specific receptors and increase the transcription of pro-inflammatory miRNA-146a and miRNA-155 by activating the NK-κB signaling pathway (Alexandrov et al., 2019), inducing the development of neuroinflammatory responses, accumulation of β-amyloid secretion, upregulation of neurogenic fibrillary tangles, neuronal and synaptic degeneration, and ultimately neuronal death, potentially leading to the development of AD (Daulatzai, 2014; Liu et al., 2019; Ortega et al., 2020).

Recent preclinical researches indicates that acupuncture may have a positive effect on the structure and function of gut microbiota and the blood–brain barrier (Dong-mei et al., 2021; Wang et al., 2023). Acupuncture may reduce levels of LPS, TNF-α and IL-1β (Zhang et al., 2022). The reduction of LPS load and systemic inflammation may play a crucial role in regulating BBB dysfunction through acupuncture (Zhang et al., 2022). Therefore, it is suggested that the gut microbiota may be a potential target for acupuncture to benignly regulate BBB dysfunction. Electroacupuncture has been found to inhibit the disruption of the blood–brain barrier in rats, thereby improving learning and memory (Lin et al., 2016). Additionally, scalp acupuncture has been shown to improve neurological function and reduce blood–brain barrier damage by up-regulating PTX3 expression and promoting occlusion band-1 (ZO-1) and occlusion protein mRNA expression (Yao et al., 2019). Electroacupuncture of GV20 and ST36 in a rat model of aging results in lower serum S100-β concentrations, suggesting that acupuncture can alleviate blood–brain barrier damage (He et al., 2021). It is well established that the blood–brain barrier, which is an essential component of the neurovascular unit, plays a critical role in lesions in the brain. Additionally, the neurovascular unit can affect brain Aβ levels (Xin, 2017; Huang Y. et al., 2020). Electroacupuncture can increase the permeability of the blood–brain barrier at specific frequencies, and this facilitates the transport of Aβ and related pro-inflammatory factors, reducing local inflammatory lesions in the brain (Zhang et al., 2018; Zhao et al., 2022a,b). Additionally, this phenomenon can aid the transport of drugs from the body’s circulation to the brain, which can have a positive impact on drug treatment for central nervous system disorders (Ma et al., 2022).

### 2.6 Effect of acupuncture on probiotics

Probiotics are a general term for beneficial bacteria, including *Lactobacillus* and *Bifidobacterium*, that regulate intestinal homeostasis and tryptophan concentration and promote the release of certain neurotransmitters (Kim et al., 2019). Studies have suggested that probiotics can influence the development and progression of AD and cognitive function (Liu C. et al., 2022; Tables 4, 5). The most common probiotics used in AD research are *Lactobacillus* and *Bifidobacterium* (i.e., *Lactobacillus Plantarum*, *Bifidobacterium bifidus*, and *Bifidobacterium Longum*; De Rijke et al., 2022). Specifically, probiotics have been shown to inhibit encephalitis by reducing IL-6, TNF-α, and CD11b (Jena et al., 2022), while the exopolysaccharide (EPS) of *Lactobacillus Plantarum* MA2 inhibits Aβ42 aggregation and amyloid-induced cytotoxic responses (Wang Y. et al., 2022). Additionally, studies suggested that *Bifidobacterium* short MCC1274 could improve cognitive dysfunction in AD by increasing the bioavailability of potential intestinal antioxidant metabolites (such as soy isoflavones and indole derivatives of tryptophan), reducing the level of Aβ42, inhibiting tau phosphorylation, improving synaptic proteins, and inhibiting microglia activation (Ohno et al., 2022; Abdelhamid et al., 2022a,b). *Bifidobacterium* short MCC1274 was also shown to reduce progression of brain atrophy and prevents cognitive impairment in elderly mild cognitive impairment (MCI) patients (Asaoka et al., 2022). Webberley et al. (2022) found that Lab4b probiotics were neuroprotective, increased mRNA expression of memory-related brain markers (including BDNF, CPLX2, and GRIA1), and reduced levels of the pro-inflammatory factor IL-10. The dietary combination of *Lactobacillus* probiotics and *Bifidobacterium Bifidus* seems to alleviate loss of brain weight in 3xTg-AD mice (Bonfili et al., 2017) and prevent cognitive decline in aging rats, AD mice, and people with AD (Akbari et al., 2016; Bonfili et al., 2017; O’Hagan et al., 2017).

**Table 4:** Relationship between probiotic and cognitive function in patients.

Study	Microorganisms	Intervention targets	Impact
Asaoka et al. (2022)	*B. breve* MCC1274	MCI patients	The probiotic treatments showed a significant improvement in the MMSE, ADAS-cog scores. Brain atrophy progression had been suppressed.
Akbari et al. (2016)	*Lactobacillus acidophilus*, *Lactobacillus casei*, *Bifidobacterium bifidum* and *Lactobacillus fermentum*	AD patient	The probiotic treated patients showed a significant improvement in the MMSE score.
Tamtaji et al. (2019)	Selenium, *Lactobacillus acidophilus*, *Bifidobacterium bifidum* and *Bifidobacterium longum*	AD patient	The probiotic and selenium treated patients showed a significant improvement in the mini-mental state examination score and cognitive function.
Kobayashi et al. (2019)	*B. breve* MCC1274	AD patient	The probiotic treated patients showed a significant improvement in the MMSE score and cognitive function.
Hwang et al. (2019)	*Lactobacillus plantarum*	MCI patient	Increased serum BDNF levels and improved cognitive function after administering DW2009.
Kim et al. (2021)	*Bifidobacterium bifidum* BGN4 and *Bifidobacterium longum* BORI	Older Adults	The probiotic increased serum BDNF levels and improved cognitive function.

**Table 5:** Relationship between probiotic and cognitive function in animal studies.

Study	Microorganism	Intervention targets	Impact
Sun et al. (2020)	*Clostridium butyricum*	APP/PS1 mice	Butyrate treatment reduces the levels of CD11b and COX-2, and suppresses phosphorylation of NF-κB p65 in the Aβ-induced BV2 microglia.
Ou et al. (2020)	*Akkermansia muciniphila*	APP/PS1 mice	Akk promoted the reduction of Aβ 40–42 levels in the cerebral cortex of APP/PS1 mice, shortened the study time and improved the completion rate in Y-maze tests.
Yang et al. (2020)	*Bifidobacterium lactis*, *Lactobacillus casei*, *Bifidobacterium bifidum* and *Lactobacillus acidophilus*	SAMP8 mice	The probiotic improved cognitive function, and that its mechanism is associated with inhibition of both TLR4-and RIG-I-mediated NF-κB signaling pathway and inflammatory responses in the APP/PS1 mouse.
Cao J. et al. (2021)	*Bifidobacterium Lactis* Probio-M8	APP/PS1 mice	Probio-M8 reduced Aβ plaque burden in the whole brain and could alleviate cognitive impairment in the APP/PS1 mouse.
Kobayashi et al. (2017)	*Bifidobacterium breve* strain A1	AD mice	The consumption of *B. breve* A1 suppressed the hippocampal expressions of inflammation and immune-reactive genes that are induced by amyloid-β.

Acupuncture has been found to affect the structure of the intestinal microbiota (Li et al., 2022). EA intervention increases the relative abundance of *Lactobacillus, Paramecium,* and *Bifidobacterium* in APP/PS1 mice and enhances their learning and memory abilities (Dong-mei et al., 2021). Additionally, electroacupuncture can up-regulate the relative gene expression of *Bifidobacterium* and *Lactobacillus* in rats (Chuan, 2021). A basic study using electroacupuncture ST36 observed an increase in the relative concentration of probiotics such as *Bacteroides*, *Clavulaceae*, and *Rhodopseudomonas*, which led to an improvement in intestinal inflammation (Huang et al., 2022). Additionally, EA significantly reduced escape latency and prolonged probing time in the target quadrant of AD rats and increased the relative DNA abundance of *Lactobacillus* and *Bifidobacteria* (Xiao et al., 2023). Furthermore, electroacupuncture has been shown to enhance the levels of *Lactobacillus,* and the pivot of intestinal alpha diversity is positively correlated with the percentage of Treg cells among CD4 cells while these cells have the ability to suppress intestinal inflammatory responses (Wei et al., 2019).

## 3 Future opportunities and challenges

The gut microbiota, the largest bacterial reservoir in the body, interacts and affects the function of various organs and systems in the body. The rapid increase of incidence and prevalence of AD represents a significant public health concern for society today. The factors involved in the development of AD are diverse and difficult to pinpoint, especially in the context of an increasingly complex society. The gut microbiota has emerged as a potential target for AD, via maintenance of gut homeostasis, activation of the immune response, modulation of inflammatory responses, and regulation of the nervous system. In this Review, we present evidence of the relationship between the gut microbiota and AD, particularly with regards to the role of acupuncture in modulating gut microbiota and discuss current challenges.

There is promising evidence that the gut microbiota impacts AD, as its structure and composition have been proven to have a functional impact on AD patients. In this context, probiotic supplementation has emerged as a potential treatment for AD. However, this therapy is not clinical evidence-based and needs to be further investigated and confirmed in a large sample in a clinical setting or a multicenter randomized controlled trial.

Acupuncture has been shown to modulate the gut microbiota. We summarized the acupoints mentioned in each study in this review (Table 6). At present, in the research related to intestinal microbiota and AD, the three meridians of ST, GV and BL are the main ones, and ST36 and GV20 are the most common acupoints combinations. However, based on our assessment of the literature, there is a lack of consistency in the choice of acupuncture points across the various studies. As the choice of the acupuncture point is the most important factor in determining the effects of acupuncture, its standardization will be essential in future research. It is also important to mention that quantification of the effect on the gut microbiota induced by the different acupuncture techniques and modalities (e.g., traditional acupuncture, warm acupuncture, auricular acupuncture, electroacupuncture) should be also standardized. Many of the studies cited in this review used electroacupuncture (EA), which is a combination of acupuncture and conventional electrotherapy. Some studies have shown no significant difference in efficacy between EA and conventional acupuncture (Amorim et al., 2022). Similarly, studies have suggested that the efficacy of EA is related to specific parameters. For example, 100 Hz EA or high-frequency acupuncture has the best analgesic effect (Zhang et al., 2023). Therefore, the future research on the role of acupuncture in AD may require the development of a comprehensive operational framework to systematically investigate its effect on the intestinal microbiota.

**Table 6:** Acupoints in each study in this review.

Study	Acupuncture points	Intervention targets	Impact
Mo et al. (2021)	GV20 + ST36 + SP6	C57BL/6 J mice	Effect of acupuncture on Alzheimer’s disease
Cai et al. (2019)	KI3	5XFAD mice	Effect of acupuncture on Alzheimer’s disease
Yu C. C. et al. (2021)	BL23 + GV20	SD rats	Effect of acupuncture on Alzheimer’s disease
He et al. (2021)	GV20 + ST36	SD rats	Effect of acupuncture on Alzheimer’s disease
Zheng et al. (2018)	LR3 + LI4	AD patients	Effect of acupuncture on Alzheimer’s disease
Tang et al. (2019)	BV20 + BL23	SPF Wistar male rats	Effect of acupuncture on Alzheimer’s disease
Xie et al. (2021)	GV20	SD rats	Effect of acupuncture on Alzheimer’s disease
Zheng et al. (2021)	GB13 + GV24	5XFAD mice	Effect of acupuncture on Alzheimer’s disease
Yang et al. (2022)	GV20 + LI4 + BL13 + BL20 + BL23 + ST36 + SP6	APP/PS1 mice	Acupuncture modulation of the gut microbiota
Wang T. et al. (2022)	GV20 + GV24 + ST36 + PC6 + GB20 + RN12	SCD patients	Acupuncture modulation of the gut microbiota
Chen D. F. et al. (2022)	GV20 + GV14 + BL23 + ST36	SD rats	Acupuncture modulation of the gut microbiota
Yu et al. (2022)	\	SD rats	Acupuncture modulation of the gut microbiota
Jiang et al. (2021)	GV20 + GV29	SAMP 8 mice	Acupuncture modulates the vagus nerve to suppress the inflammatory response of the intestinal microbiota
Zhang et al. (2022)	GV20 + GV29 + ST36	APP/PS1 mice	Acupuncture modulates the vagus nerve to suppress the inflammatory response of the intestinal microbiota
Hao et al. (2022)	ST36 + GV20 + GV29	APP/PS1 mice	Acupuncture modulates the vagus nerve to suppress the inflammatory response of the intestinal microbiota
Wang et al. (2018)	CV12 + BL21 + CV12 + BL21	SD rats	Acupuncture modulates the vagus nerve to suppress the inflammatory response of the intestinal microbiota
Li Y. et al. (2021)	ST36	SD rats	Acupuncture modulates the vagus nerve to suppress the inflammatory response of the intestinal microbiota
Wang et al. (2019b)	ST36	SD mice	Acupuncture modulates the vagus nerve to suppress the inflammatory response of the intestinal microbiota
Wang et al. (2019b)	ST36	WT mice	Acupuncture modulates the vagus nerve to suppress the inflammatory response of the intestinal microbiota
Zhang Z. et al. (2021)	ST36	BALB/c mice	Acupuncture modulates the vagus nerve to suppress the inflammatory response of the intestinal microbiota
Yang F. et al. (2021)	ST36	C57BL/6 J mice	Acupuncture modulates the vagus nerve to suppress the inflammatory response of the intestinal microbiota
Du et al. (2013)	ST36	SD rats	Acupuncture modulates the vagus nerve to suppress the inflammatory response of the intestinal microbiota
Yang F. et al. (2021)	ST36	Wistar rats	Modulation of autoimmunity by acupuncture
Bai et al. (2021)	PC6	SD rats	Modulation of autoimmunity by acupuncture
Ma et al. (2021)	GV24 + ST25 + LI11 + ST37	C57BL/6 J mice	Modulation of autoimmunity by acupuncture
Jang et al. (2020)	ST36 + GB34	C57BL/6 J mice	Modulation of autoimmunity by acupuncture
Zhou et al. (2022)	SP6 + ST36	SD rats	Modulation of autoimmunity by acupuncture
Sun et al. (2022)	GV20	C57BL/6 J mice	Modulation of autoimmunity by acupuncture
Deng et al. (2022)	ST36 + GV20	C57BL/6 J rats	Modulation of autoimmunity by acupuncture
Srinivasan et al. (2016)	GV20 + GV29	SAMP8 rats	Modulation of autoimmunity by acupuncture
Cao et al. Y. (2021)	ST36 + GV20	Wistar rats	Modulation of autoimmunity by acupuncture
Chuan (2021)	ST36 + GV20	SD rats	Effect of acupuncture on probiotics
Huang et al. (2022)	ST36	SD rats	Effect of acupuncture on probiotics
Xin (2017)	GV20 + KI1	APP/PS1 mice	Effect of acupuncture on probiotics
Ma et al. (2022)	GV20 + GV26	SD rats	Effect of acupuncture on probiotics

In addition, there is scarce research on the role of specific acupuncture points on changes of microbiota and on the role of probiotics and therefore further investigations are needed.

The blood–brain barrier, an essential component of the neurovascular unit, is essential to maintain brain homeostasis. Based on evidence from the literature, acupuncture appears to repair a damaged blood–brain barrier. However, the exact mechanism underlying this effect is unknown and the number of studies on this topic is insufficient to draw robust conclusions. Similarly, no evidence exists to allow conclusions on whether acupuncture can prevent or repair the natural breakdown of the blood–brain barrier, which is particularly important in aging and AD. As the blood–brain barrier allows exchange of substances within and outside the brain, it can also transport harmful substances and nutrients into the brain as a result of an inflammatory response. Acupuncture can increase the permeability of the blood–brain barrier and reduce the central inflammatory response. However, as noted above, further research on research is needed.

We reviewed the recent evidence related to acupuncture and modulation of the gut microbiota in AD. We mainly included studies published in past 5 years, which reports promising results. However, there are inevitable limitations. There was high heterogeneity in the animal studies included due to differences in intervention protocols, intervention targets, feeding methods, and outcome measures. There were differences in the studies with regards to the type of acupuncture intervention, techniques, choice of acupuncture points, effects (i.e., sensations such as acid, numbness, swelling, pain, etc.) and outcomes. We summarised the characteristics of studies included in this review in Supplementary Tables S1, S2.

In conclusion, targeting the gut microbiota offers promising potential therapeutic targets for AD, but further research is needed before these findings can have clinical impact in patients with AD.

## Author contributions

LY: Writing – original draft, Writing – review & editing, Formal analysis, Methodology, Software, Validation. HL: Conceptualization, Formal analysis, Methodology, Project administration, Validation, Writing – review & editing. YQ: Conceptualization, Formal analysis, Methodology, Project administration, Validation, Writing – review & editing. QL: Graphics. SC: Conceptualization, Writing – review & editing. BD: Funding acquisition, Writing – review & editing. YW: Funding acquisition, Writing – review & editing. MW: Conceptualization, Supervision, Writing – review & editing. TY: Supervision, Writing – review & editing.
