# Supplementary material for: Acupuncture modulates the gut microbiota in Alzheimer’s disease: current evidence, challenges, and future opportunities
Source: Front Neurosci. 2024 Mar 1;18:1334735. doi: 10.3389/fnins.2024.1334735 (PMC10940355; doi:10.3389/fnins.2024.1334735)
Supplement: Supplementary file 1 [file Table_1.DOCX]

We described characteristics of included animal studies in the Supplementary Table 1 referring to the new ARRIVE guidelines, as well as characteristics of included human studies in Supplementary Table 2.

Supplementary Table 1 Characteristics of included animal studies referring to the new ARRIVE guidelines.

| Sample size | Treatment | Treatment course | Housing and husbandry conditions | Experimental outcomes | Refs. |
| --- | --- | --- | --- | --- | --- |
|  |  |  |  |  |  |
|  |  |  |  |  |  |
| C57BL/6J mice | EA | the mice were followed by EA treatment (2 / 15 Hz, 1 mA, 30 min) at the Baihui point (DU-20), Zusanli point (ST-36), and Sanyinjiao point (SP6) once daily for 7 days by a HANS-200A instrument. | All animals were performed in accordance with the Care and Use of Labo ratory Animals (Committee for the Update of the Guide for the Care and Use of Laboratory Animals, 2011). | PICK1,TLR4, ,TLR4-related proteins | Y. Mo, L. Wang, M. Ren et al.,2021 |
| ADLPAPT rats | Fresh faecal matters of WT mice were orally provided to  ADLPAPT mice | 16 weeks | / | spontaneous alternation in Y-maze, contextual fear-con-  ditioning, open-field test | M.S. Kim, Y. Kim, H. Choi et al.,2020 |
| SAMP8 mice (n=40） | EA | The disposable sterile acupuncture needles (0:3mm× 13mm) were used to puncture the Yintang (GV29) with transverse puncturing to the nasal root direction at a depth of 10mm, and the bilateral Yingxiang (LI20) with shallowly puncturing towards the interior and superior at a depth of 2mm. The bilateral Yingxiang (LI20) was stimulated with HANS-200 electric stimulator for 10 minutes (current intensity: 1.5 mA, wave: 15 Hz). | at a controlled temperature (22 ± 2°C), 55% humidity, and under a 12h dark/light cycle, with sterile drinking water and a standard pellet diet available ad libitum. | Aβ,SYP,p38MAPK | Y. Wang, Q. Wang, B. Ren et al.,2020 |
| 5XFAD mice | EA | Acupuncture needles were inserted at a depth of 2 mm, and electrical stimulation pulses were applied for 15 min (1 mA, 2Hz) at the bilateral KI3 acupoints. The anode and cathode of the electrical stimulator were connected to the two acupuncture needles.. | Four animals per cage were housed in an environment with a temperature of 21 ± 3 °C and humidity of 50 ± 10% with a 12-h light/dark cycle (light on 07:00–19:00 h). Access to water and food was ad libitum. | synaptophysin,postsynaptic density-95 protein,Aβ | M. Cai, J.H. Lee, E.J. Yang,2019 |
| Male C57BL/6J mice | injection of three SCFA molecules /*Systemic capsaicin treatment* | Day1Day3Day5 injection capsaicin | Controlled temperature (23 ± 1 °C), humidity (55% ± 5%), and lighting (light phase 7:30-19:30).Food and water were available ad libitum. | Immunohistochemical detection of pERK1/2 in NG and medial NTS  Measurement of [Ca2+]i in single NGN | C. Goswami, Y. Iwasaki, T. Yada,2018 |
| Male Sprague-Dawley rats | abdominal surgery | RX-77368 (50 ng/rat) or saline was injected followed, 1 h later, by laparotomy and small intestinal/cecal manipulation. The sham group had anesthesia alone. | Male Sprague-Dawley rats (Harlan, San Diego, CA) weighing 280–310 g were housed under controlled conditions of temperature (22–24°C) and light (from 6:00 am to 6:00 pm) for at least 1 wk before the experiments. | gastric emptying (GE) and the inflammation in gastric corpus were determined/ MHCII (M1 marker) and CD206 (M2 marker) | P.Q. Yuan, Y. Taché,2017 |
| 5XFAD mice and co-housed WT mice | GV-971 | one month/ twice per day | All the mice were maintained in a room at 23 °C under a 12-hour (h) light-dark cycle. | Morris water maze (MWM) and Y maze tests/Faecal sample DNA extraction, PCR amplification and sequencing | X. Wang, G. Sun, T. Feng et al.,2019 |
| C57BL/6J mice | they were either placed on the NAD precursor-deficient diet, or the NA-supplemented diet, or were continued on the NAD precursor-sufficient diet. | Five days prior to colonization, 8-week-old male mice were switched to the NAD precursor-sufficient diet; 2 days later, they were either placed on the NAD precursor-deficient diet, or the NA-supplemented diet, or were continued on the NAD precursor-sufficient diet | Germ-free C57BL/6J mice were reared in plastic flexible film gnotobiotic isolators (Class Biologically Clean) at 23°C under a strict 12-h light cycle (lights on at 0600h, off at 1800h) | NADase activity | J.S. Weagley, M. Zaydman, S. Venkatesh et al.,2022 |
| APP/PS1 mice  (n=35) | the gut microbiota from AD patients were transplanted into APP/PS1 double transgenic mice | Behavioral tests were carried out 0, 7, 14, 21, 28  days after transplantation.  For navigation test, each mouse was examined four times a day (two times in the morning  and another two times in the afternoon) | All the animal experimental procedures were carried out in accordance with the animal  ethics . | the expression of NLRP3  and the expression levels of inflammatory factors in peripheral blood/the peripheral  blood inflammatory factors, IL-1β, IL-18 and TNF-α | H. Shen, Q. Guan, X. Zhang et al.,2020 |
| 3×Tg mice | recolonisation of germ-free 3×Tg mice with fecal samples from both patients with AD and age-matched healthy donors | / | All the animal experimental procedures were carried out in accordance with the animal  ethics . | amyloid-β/ neurofibrillary tangles pathology | C. Chen, J. Liao, Y. Xia et al.,2022 |
| 3×Tg-AD mice and C57BL/6J mice | FMTs were administered orally | A single 200 μL bolus of the prepared microbiota suspension was administered by oral gavage to each recipient C57BL/6J mice (wild-type, WT) at 24 h after TBI. | The Institutional Animal Care and Use Committee (IACUC) approved all animal studies and breeding protocols at Houston Methodist Research Institute. | characterized the microbiota composition of the fecal samples by full-length 16S rRNA gene sequencing analysis | S. Soriano, K. Curry, Q. Wang et al.,2022 |
| C57BL / 6J mice | infusion of the serum protein albumin into the young rodent brain | / | Animals were housed with a 12-hour light/12-hour dark cycle with food and water available ad libitum | TGFβ signaling | V.J. Senatorov, A.R. Friedman, D.Z. Milikovsky et al., 2019 |
| C57BL/6J mice | intervention using probiotics/ antibiotic treatment | after eight months of WD-feeding, mice were randomly assigned to two groups to receive either B. infantis (109 CFU/mL, daily oral) or PBS (control) for two months/7-month-old WD-fed mice received without or with broad-spectrum coverage antibiotics (ABX) consisting of Ampicillin (1 gm/L), Metronidazole (1 gm/L), Vancomycin (0.5 gm/L), and Neomycin (1 gm/L) in drinking water for 3 months while mice continued to be fed by WD. | housed in steel micro-isolator cages at 22 °C with a 12-h light/dark cycle. To study the dietary effect, mice were provided a control healthy diet (CD; 5.2% fat, 12% sucrose, and 0.01% cholesterol, w/w, TD. 140415) or a WD (21.2% fat, 34% sucrose, and 0.2% cholesterol, w/w; TD. 140414) (Envigo, Indianapolis, IN, USA) after weaning (3 weeks, more than 4 mice per group were used in each experiment) | IL6, TNFα, CD11b, LTP, PSD95, BDNF | P.K. Jena, T. Setayesh, L. Sheng et al.,2022 |
| AppNL - G - F） mice | orally supplemented with B. breve MCC1274 or saline | 4 month | The plasma samples were obtained from studies performed at Nagoya City University which were approved by Nagoya City University Institutional Care and Use of Laboratory Animals committee to understand the mechanisms of action and explore this probiotic pharmaceutical potential. | plasma metabolites/The levels of soy isoflavones (e.g., genistein) and indole derivatives of tryptophan (e.g., 5-methoxyindoleacetic acid) | K. Ohno, M. Abdelhamid, C. Zhou et al.,2022 |
| App^NL-G-F^ mice (n=52) | the vehicle group （received saline）/the probiotic group（was supplemented with B.breve MCC1274 (1×109 cfu/5.56 mg/200μl saline/mouse) via oral gavage）. | 5 times a week, over the course of 4 months | All the experiments were carried out in conformity with the National Institute of Health Guide for the Care and Use of Laboratory Animals and were approved by Nagoya City University Institutional Care and Use of Laboratory Animals committee. | Aβ levels, Aβ fibril, IL-1β、IL-6、TGF-β1 | M. Abdelhamid, C. Zhou, K. Ohno et al.,2022 |
| C57BL/6J mice (n=40) | One group received saline and the other group was administered 1 × 10^9^ cfu/6.25 mg/200 μL saline/mouse/day of B. breve MCC1274 via oral gavage. | 5 times a week, over the course of 4 months | The mice were housed under controlled humidity and temperature conditions with a 12-h light–dark cycle and ad libitum access to water and pellet food (Oriental Yeast Co., Tokyo, Japan). | Aβ levels, amyloid precursor protein (APP), APP processing enzymes, phosphorylated tau, synaptic protein levels, glial activity, and cell proliferation in the subgranular zone of the dentate gyrus. | M. Abdelhamid, C. Zhou, C.G. Jung et al.,2022 |
| 3xTg-AD mice (n=20) | one group received high fat diet alone for 12 weeks (termed the “Control” group) and the second group received HFD supplemented with the Lab4b probiotic providing a minimum of 5 × 108 colony forming unit (cfu)/mouse(termed the “Probiotic” group) | Once a day for 12 weeks | Mice were housed in scantainer vented cages (2–4 mice per cage) in a light and temperature-controlled environment (12 h on/off light at 22°C) and had ad libitum access to all diet and water. | Behavioural Testing,  Hippocampal Dendritic Spines, , TC, HDL， LDL, vLDL, TG, IFN-γ，IL-2，IL-4，IL-5，IL-6，IL-10，IL-1β， KC / GRO， TNF-α, microbiota | T.S. Webberley, G. Masetti, R.J. Bevan et al.,2022 |
| 64 3xTg-AD mice and 64 wt mice | A treated group (administered for 4 months with SLAB51 in water) and a control group (administered with water). Simultaneously, wt mice were divided into wt control and wt treated groups. | 4 months | Mice were housed in plastic (Makrolon) cages (4 animals per cage) in a temperature controlled room (21 ± 5 °C) and 60% humidity on 12 h light/dark inverted cycle (light was switched on at 8:00 P.M.) and maintained on laboratory diet (Mucedola, Italy) with water ad libitum. | the open field test, the novel-object recognition test, the passive avoidance test, the elev*ated plus maze ,* IL1α，IL1β，IL2，IL12，IL17，IFNɣ, TNFα, IL4，IL6，G-CSF, GM-CSF, *SCFA), gut hormone,* Aβ, proteasomal and autophagic proteolytic (amyloid oligomers, ubiquitinated proteins, p53, p27, the autophagy related proteins Beclin-1, p62, LC3-II). | L. Bonfili, V. Cecarini, S. Berardi et al.,2017 |
| adult male Lister Hooded rats (n=32) | control group /diet supplemented group | / | All experiments were performed in accordance with the UK Animals (Scientific Procedures) Act (1986) and associated guidelines, as well as European Union directive 2010/63/EU. | MWM，Object recognition memory，Object-in-place memory，Object temporal order，H NMR spectroscopy | C. O'Hagan, J.V. Li, J.R. Marchesi et al.,2017 |
| 20 APP/PS1 mice and 10 WT mice | CB/CMC Na/CMC Na | daily for 4 weeks | All mice were raised at the Experimental Animal Center of Wenzhou Medical University in a controlled temperature (22 ± 1 °C) environment with relative humidity (65 ± 5%). The mice were housed under a light/dark cycle, and water and food were available ad libitum during the experiment. | MWM, FJC-positive cells, GM, Aβ42, IL-1β, TNF-α, butyrate, microbiota(DNA, 16S rRNA), BV2 microglia, CD11b, COX-2, NF‐κB-p65 | J. Sun, J. Xu, B. Yang et al., 2020 |
| 40 APP/PS1 mice and 12 WT mice | AKK | Once a day for 6 months | / | MRI, Aβ, Chol, TG, ALT, AST, DAO, CUSABIO, Open-field and Y-maze tests, blood glucose, microbiota | Z. Ou, L. Deng, Z. Lu et al.,2020 |
| 24 SAMP8 mice and 12 SAMR1 mice | ehicle (water)/ProBiotic-4 (2 × 109 CFU) | once daily for 12 weeks | All of the animal studies were conducted in accordance with the Regulations of Experimental Animal Administration issued by the State Committee of Science and Technology of the People's Republic of China. | the grading score system and Y-maze test, BBB, microbiota(DNA, 16S rRNA), Iba-1, GFAP, TNF-α, IL-6, LBS, 8-OHdG, TLR4, SYN, γ-H2AX, β-actin, RIG-I, NF-κB P65, lamin B, claudin-5, occludin, ZO-1 , VE-cadherin | X. Yang, D. Yu, L. Xue et al.,2020 |
| APP/PS1 mice | Probio-M8 （1×109CFU/mL）/saline water | 45 days | All the mice were maintained at the Institute of Laboratory Animal Science in Jinan University (Guangzhou, China) under a controlled environment (temperature: 23 ± 2 °C; humidity: 45 ± 10%; 12-h light/12-h dark cycle). Mice were fed regular chow (purchased from BEIJING HFK BIOSCIENCE CO., LTD, 1035) and sterile water. | Y-maze test, microbiota(DNA, 16S rRNA), Aβ | J. Cao, W.K. Amakye, C. Qi et al.,2021 |
| ddY mice | B. breve A1/sodium acetate/ donepezil | 11 days | All procedures were performed in accordance with the National Institutes of Health guidelines for the use of experimental animals. | Y-maze test, microbiota(DNA, 16S rRNA), SCFA | Y. Kobayashi, H. Sugahara, K. Shimada et al.,2017 |
| 40 SAMP8 mice (n=40) and SAMR mice (n=10) | ACU | Once a day for 28 days | All mice were raised in the animal room of the First Affiliated Hospital of Tianjin University of Traditional Chinese Medicine under the following conditions: natural light, free feeding and drinking, temperature of 25 ℃, relative humidity of 50%, noise lower than 80dB, and the animal room environment and cage were kept clean and breathable. | Morris water maze test , Aβ42, p-tau,  RhoA, ROCK, F-actin , p-cofilin | W. Yu, Z. Lan, S. HuiY et al.,2021 |
| SD rats (n=72) | ACU | Once a day for 8 weeks | All rats were housed (n = 4 per cage) at 20 ± 2°C and a relative humidity of 50 ± 10% under a 12-hour light/dark cycle (lights on at 8:00 a.m.), with free access to food and water. | Morris water maze test, tau-5，PHF-1, tau-pS262, GSK-3β, DNMT1 | C. He, Z.S. Huang, H.R. Chen et al.,2020 |
| SD rats (n=72) | ACU | Once a day for 8 weeks | All rats were housed (n = 4 per cage) at 20 ± 2°C and a relative humidity of 50 ± 10% under a 12-hour light/dark cycle (lights on at 8:00 a.m.), with free access to food and water. | Morris water maze test, tau-5，PHF-1, tau-pS262, GSK-3β, DNMT1 | C. He, Z.S. Huang, H.R. Chen et al., 2020 |
| SD rats (n=36) | EA | 5 Hz in frequency, 1 mA in intensity, 20 min each intervention, once a day for 8 weeks | During the experiment, the rats were treated in strict accordance with the Guidelines on Treating Experimental Animals issued by the Ministry of Science and Technology of the People's Republic of China in 2006 Guiding Opinions. | the Morris water maze test.，NLRP3, Caspase-1， IL-1β protein，MG | C.C. Yu, C. He, Du YJ et al.,2021 |
| SPF Wistar male rats (n=40) | ACU | 6 days per week for 2 weeks | The whole experiment process strictly followed the relevant provisions of the Guiding Opinions on Treating Experimental Animals Properly issued by the Ministry of Science and Technology of the People's Republic of China. | MWM, the ultrastructure changes of neurons and astrocytes, hippocampal dentate gyrus | S.H. Tang, Du YJ, Y.M. Tao et al., 2019 |
| SD rats (n=20) | ACU | 6 days per week for 3 weeks | / | MWM, DG, CA1, GFAP, Iba1, iNOS, Arg1, IL-1β，TNF-α, IL-6, IL-4, IL-10, p65, Stat6 | L. Xie, Y. Liu, N. Zhang et al., 2021 |
| 5XFAD mice and WT mice | EA | Needle handles were connected to an electrical stimulator and electrical stimulation pulses were applied for 15 min (0.3 mA, 2 Hz). EA treatment was performed 5 times per week for 4 weeks. | All rats were maintained at 23 ± 2°C and 60 ± 15% relative humidity with free access to feed and water. | NWM, FC, APP/Aβ, LC3B-II, SQSTM1, MTORC1，MAPK1, AKT | X. Zheng, W. Lin, Y. Jiang et al.,2021 |
| C57BL/6J mice (n=90) | EA | Ⅰcontrol group/DSS-induced colitis (DSS) group/DSS with sham EA (DSS + SEA) group (acupuncture only without electric current)/DSS with low-frequency EA (DSS + LEA) group (10 Hz, 1 mA)/DSS with high-frequency EA (DSS + HEA) group (100 Hz, 1 mA) for 84 days. | All animals were housed in a specific pathogen-free facility in the laboratory animal center of Tongji Medical College (22 °C, 12-h light/12-h dark cycle) with free access to food and sterile water. | TNFα, IL1β, IL6 , iNOS, IL10 , ZO-1, [Occludin](https://www.sciencedirect.com/topics/medicine-and-dentistry/occludin), E-Cadherin, MUC2, MAPK, [gut microbiota](https://www.sciencedirect.com/topics/pharmacology-toxicology-and-pharmaceutical-science/intestine-flora), FMT，DAI | L. Wang, J. An, S. Song et al.,2020 |
| APP/PS1 mice (n=24) and 6 C57BL / 6J mice (n=6) | ACU | 20 minutes per day and 5 days per week | To reduce external interference, mice were housed separately in standard mouse cages under constant temperature (23 ± 2°C) and constant humidity (40%-60%), with free access to water and food. The study was conducted in strict accordance with the regulations of the Animal Ethics Committee (20200811-01). | MWM, Aβ, tau, IL-1β，IL-10，LPS, TNF-α, [gut microbiota](https://www.sciencedirect.com/topics/pharmacology-toxicology-and-pharmaceutical-science/intestine-flora) | B. Yang, M. He, X. Chen et al.,2022 |
| SD rats (n=68) | EA | EA (10 Hz/50 Hz) was conducted for 30 min, once daily for 4 consecutive weeks | The room temperature is 23 ℃, the humidity is 52%~60%, and the light/dark cycle is 12h/12h. | MWM, IL-1β, IL-18, [gut microbiota](https://www.sciencedirect.com/topics/pharmacology-toxicology-and-pharmaceutical-science/intestine-flora)([16S rRNA](https://www.sciencedirect.com/topics/medicine-and-dentistry/rna-16s), Catabacter, Robinsonella, Desulfovibrio), histopathological changes of the intestinal mucosa, ultrastructural changes of hippocampal neurons | D.F. Chen, H. Zhang, J.Y. Xie et al.,2022 |
| SD rats (n=60) | EA | GV20 point was punctured at an angle of 15 degrees for 2 mm depth and ST36 point was punctured perpendicularly for 4 mm. Continuous wave with frequency of 50 Hz and the intensity of 1 mA was selected in EA treatment for 20 minutes in accordance | The rats were kept in temperature-and light-regulated cage rooms with 12/12 hr light/dark cycle at the 20±2 °C temperature. Food and water were provided ad libitum. | MWM, ZO-1, TLR4, NF-κB, LPS, DAO, S-100β, Iba-1, [gut microbiota](https://www.sciencedirect.com/topics/pharmacology-toxicology-and-pharmaceutical-science/intestine-flora)(DNA, Lactobacillus,  Bifidobacterium, Bacteroides fragilis, Escherichia coli) | C. He, Z.S. Huang, C.C. Yu et al.,2021 |
| SD rats (n=24) | ACU | once a day for7 consecutive days | Animals were housed individually in a temperature-controlled room (24 ± 2°C) under a 12/12-h light-dark cycle. The rats were allowed free access to food and water throughout the experiment. | Open field test, 5-HT，NE，Ach，GABA, cAMP, [gut microbiota](https://www.sciencedirect.com/topics/pharmacology-toxicology-and-pharmaceutical-science/intestine-flora)(DNA, [16S rRNA](https://www.sciencedirect.com/topics/medicine-and-dentistry/rna-16s)), GAT-1 | H. Yu, H. Yu, L. Si et al.,2022 |
| SAMP8 mice (n=24) and SAMR1 mice (n=8) | EA | GV20 and GV29 were chose for electroacupuncture for 15min per day, with transverse puncturing at a depth of 4–5 mm using disposable sterile acupuncture needles. The needles were taped and connected to electroacupuncture device with the sparse wave at 2 Hz, 2 V, and 0.1 mA. | All procedures were complied with the ARRIVE guidelines were performed according to the guidelines of the National Institutes for Animal Research (ID: BUCM-4-2018111701-4045). | MWM, [gut microbiota](https://www.sciencedirect.com/topics/pharmacology-toxicology-and-pharmaceutical-science/intestine-flora)(DNA), Dentate gyrus of hippocampus, IL-1β，IL-6, TNF-α | J. Jiang, H. Liu, Z. Wang et al.,2021 |
| APP/PS1 mice (n=80) and C57BL/6J (n=20) | ACU | 45 days | The mice weighed 30.0 ± 2.0 g and were 6 months old. Water and food were provided ad libitum. | MWM, [gut microbiota](https://www.sciencedirect.com/topics/pharmacology-toxicology-and-pharmaceutical-science/intestine-flora)([16S rRNA](https://www.sciencedirect.com/topics/medicine-and-dentistry/rna-16s)), BBB, LPS, TNF-α, IL-1β | Y. Zhang, N. Ding, X. Hao et al.,2022 |
| APP/PS1 mice (n=72) and C57BL/6J mice (n=18) | ACU | 45 days | all experimental procedures complied with the ARRIVE guidelines and were performed according to the guidelines of the National Institutes for Animal Research (ID: bucm-4-2021102701-4032). | MWM, [gut microbiota](https://www.sciencedirect.com/topics/pharmacology-toxicology-and-pharmaceutical-science/intestine-flora)([16S rRNA](https://www.sciencedirect.com/topics/medicine-and-dentistry/rna-16s))，GFAP，LPS，occludin，ZO-1，FITC，pithelial intestinal tight junctions，TNF-α， | X. Hao, N. Ding, Y. Zhang et al.,2022 |
| SD rats (n=30) | ACU | once a day for 7 days | The disposal of animals during the experiment was in line with that of the Ministry of Science and Technology of the People's Republic of China in 2006 Guiding Opinions on Treating Experimental Animals. | NO, Gastric motility, Gastric motility, subunit NR1 of NMDAR | L. Wang, G. Shen, H. Wang et al.,2018 |
| SD rats (n=84) | EA | EA stimulation of both limbs was performed by inserting unipolar stainless steel needle electrodes, with a diameter of 0.25 mm and length of 12 mm, at ST36 bilaterally, advancing them to a depth of 7 mm and connecting them to an EA apparatus. Electrical stimulation was applied using a continuous pulse mode for 30 min with an intensity of 2 mA, pulse width of 50 ms and frequency of 10 Hz. | The experiment was conducted in compliance with the Guide for Care and Use of Laboratory Animals of the National Research Council, China. | DA, TNF-α, MPO, MDA | ]Y. Li, G. Xu, S. Hu et al.,2021 |
| SD rats (n=34) | EA | N/A /7days/90 min | After a 7-day acclimation period, they were housed in individual plastic cages placed in rooms with temperature of 22–23°C, humidity of 40–50%, and on a12:12-h light-dark cycle. Water and food were available ad libitum. Female rats were excluded from the study to avoid a possible compounding effect due to female hormones. | WGTT, dCTT, HRV/WGTT, dCTT, HRV, /gastric emptying, intestinal transit | X. Wang, B. Yang, J. Yin et al.,2019 |
| Wild-type mice | EA | Electrical stimulation (current, 2 mA; frequency, 2/15 Hz) was applied for 20 min long. | All animal protocols were reviewed and approved by the Institutional Animal Care and Use Committee of Nanjing University of Chinese Medicine and were performed in accordance with the guidelines for animal research. | Heart Rate Variability Analysis  （LF/HF），TNF-α, IL-1β, IL-6，MPO，CD11bLy6Gcells，CD11bF4/80，α7nAChR | L. Zhang, Z. Wu, J. Zhou et al.2021 |
| C57BL/6 mice | EA | The electrical stimuli were administered using a HANS-200A Acupuncture Point Nerve Stimulator (Nanjing, China) at an intensity of 1 mA, 10 Hz, plus width 0.4 ms, 20 min, which, in our experience, is the threshold to detect a muscle twitch. | The principles of laboratory animal care were followed; the Institutional Animal Care and Use Committee (IACUC) approved animal activities under the approval number BUCM-4-2018101901-4009. | α7nAChR, JAK2/STAT3 signaling pathway, GABA receptor | N.N. Yang, J.W. Yang, Y. Ye et al.,2021 |
| SD rats | EA | ST36 acupuncture point was punctured with a depth of 7 mm, and then the needle was connected with an electro-acupuncture apparatus. The electric current with the intensity of 2 mA and 2-100Hz was continued for 1.5 h immediately after hemorrhage. | Rats were acclimatized for a while in mesh cages in a temperature-controlled room with a 12-h light-dark cycle in the animal quarter of our laboratory and fasted overnight, but allowed free access to water until 4 h before surgery. | IL-6,intestinal permeability | Du MH, H.M. Luo, S. Hu et al.,2013 |
| Wistar rats  (n=132) | ACU | Each rat was immobilized using a soft cloth. ST36 is located 3-4 mm below and 1-2 mm lateral to the midline of the knee, and the disposable acupuncture needles (diameter = 0.35 mm, length = 25 mm,) were vertically inserted into bilateral ST36 to a 3 mm depth of rats in the MA group. After deqi, the needles were rotated at a rate of 3 spins bidirectionally. | All rats were allowed to acclimatize for one week prior to the experiment and were housed in a controlled environment (12 h light/dark cycle, 22 ± 2°C, 55 ± 5% relative humidity) with free access to food and water. All rats used in the present study were healthy and have never been used in other research procedures. | IL-1β,IL-α,TNF-α,IL-18,IL-6,IL-7,  CXCL 1,MCP-1,MIP 3 α,RANTES,MIP-1α | F. Yang, Y. Gong, N. Yu et al.,2021 |
| SD rats (Sham (n = 6), Esham (n = 7), MIRI (n = 6), EMIRI (n = 7)） | EA | The needles were connected to Han's Acupoint Nerve Stimulator and stimulated with an intensity of 2 mA and a frequency of 2/15 Hz. | All procedures were conducted in accordance with the National Institutes of Health's Guide for the Care and Use of Laboratory Animals. | LPS,TLR4,IL-6 | H. Bai, R.J. Gu, L.Y. Chen et al.,2021 |
| C57BL/6 mice (n=24) | EA | The needles were then connected to an acupuncture point nerve stimulator, and stimulation with a frequency of 2 Hz and an intensity of 1 mA was administered for 10 min. | All mice were housed individually at 24 ± 2 °C under a 12/12 h light/dark cycle with free access to food and water for at least 7 days before the start of the experiments. | tyrosine hydroxylase,Iba-1,a-synu-  Clein,ZO-1,occludin,IL-1b,IL-6,TNF-α,ChAT,TH,nNOS | X. Ma, Q. Wang, W. Yuan et al.,2021 |
| C57BL/6 mice (the control group ( n = 6), the MPTP group (n = 10), the MPTP + Acu group (n = 9), the MPTP + Non-acu (n = 5)） | ACU | Acupuncture needles on the acupoints GB34 and ST36 were inserted bilaterally to a depth of 3 mm at each acupoint, turned at a rate of two spins per second for 30 s and then removed immediately. Acupuncture treatments were performed at 2 h after MPTP treatment (1st–5th day), and [at 10](https://www.sciencedirect.com/topics/neuroscience/dihydrotachysterol):00 AM (6th–12th day). | Seven-week-old male C57BL/6 mice (weight, 23–25 g; Central Laboratories Animal Inc., Seoul, Republic of Korea) were housed individually at 24 ± 2 °C under a 12/12 h light/dark cycle with free access to food and water for at least 7 days before the start of the experiments. | TH,GFAP,Iba-1,NF-κB,TNF-α | J.H. Jang, M.J. Yeom, S. Ahn et al.,2020 |
| SD rats（cintrol (n=8),TNBS(n=8), TNBS + EA (n=8)） | EA | ST36 and SP6 were selected as the acupuncture point and inserted bilaterally to a depth of approximately 7 mm, 5 mm after disinfection, respectively. The parameters were implemented with 5 Hz and 0.2 mA in the TNBS + EA group. For the sham EA, at the same treatment points, the TNBS group received electrodes without needle penetration into the skin and current was set to 0 while the control group only received topical disinfectant. | All rats were housed in the Laboratory Animal Center of Zhejiang Chinese Medical University at 21 ± 2°C and 55 ± 10% humidity with free access to autoclaved chow and water. After acclimation to the environment for 7 days. | GABA,ZO-1,NF-κB,NLRP 3,IL-1β,HPA | F. Zhou, H. Jiang, N. Kong et al.,2022 |
| C57BL / 6J mice | EA | Mice were immobilized and the stainless‐steel needle (0.16 mm × 7 mm) was inserted into the GV20 vertically with a depth of 2 mm. The parameters of EA were set as follows: intensity 0.5 mA and frequency: 2 Hz. The EA stimulation was performed for 20 min each time and twice daily for 7 days. | The experimental procedures were carried out according to the “Guide for the Care and Use of Laboratory Animals” published by the NIH. | IL-1β,IL-6,NLRP 3,NF-κB | L. Sun, Y. Yong, P. Wei et al.,2022 |
| C57BL / 6J rats (n=50) | EA | EA treatment was performed three times at 0 h, 24 h and 48 h, respectively after reperfusion. For the EA acupuncture group, acupuncture needles were stuck into the mice at GV20 and ST36 at a depth of 2–3 mm. EA stimulation was carried out for 30 min at 2 Hz frequency with continuous waves using an electroacupuncture device . | All experimental protocols were conducted according to the guidelines of the National Institutes for Animal Research and approved by the Ethics Committee for Animal Experimentation of Peking Union Medical College Hospital of the Chinese Academy of Medical Sciences (reference no. XHDW-2019-056). | Iba 1+,IL-33/ST 2 | P. Deng, L. Wang, Q. Zhang et al.,2022 |
| SAMP8 rats(control(n=10),AD(n=10),drug(n=10),EA(n=10)） | EA | Mice were immobilized in mouse bags. GV20 and GV29 were chosen for electroacupuncture for 15 min per day. The needles were taped and connected to the HANS-LH202 electroacupuncture device with the sparse wave at 2 Hz, 2 V, and 0.1 mA | All procedures complied with the Animal Research: Reporting of In Vivo Experiments (ARRIVE) guidelines and were performed according to the guidelines of the National Institutes for Animal Research. | TREM 2,IL-1β,TNF-α | Y. Li, J. Jiang, Q. Tang et al.,2020 |
| Wistar rats | ACU | The needles were perpendicularly inserted to a 5 mm depth and retained in place for 10 min.  Acupuncture was performed once daily for 2 weeks (continued for 6  days and 1 day of rest) beginning from the third day after surgery. | Wistar rats (260-300g, 7-8 weeks Journal Pre-proof Journal Pre-proof 4 old, male) were housed at constant room temperature (22 °C) and humidity (60-70%) under a 12 h light–dark cycle and standard rodent diet and tap water. | α 7nAChR,JAK2/STAT3 | Y. Cao, L. Wang, L.T. Lin et al.,2021 |
| SD rats | EA | The needles were then connected to an acupuncture point nerve stimulator, and stimulation with a frequency of 1/50 Hz and an intensity of 1 mA was administered for 20 min. | All animal experiments conducted in this study strictly follow the Guiding Opinions on the Good Treatment of Laboratory Animals issued by the Ministry of Science and Technology of the People's Republic of China | IL-1β,LPS,IL-6,TNF-α,microbiota | HeChuan, 2021 |
| SD rats  （NC (n=10); CAG (n=10); EA (n=10); Vit (n=10) | EA | The two needles were connected with a G6805-I Electroacupuncture Stimulator or 30 min, alternating between the left and right side acupoints. Output parameters were sparse and dense waves (sparse wave 4 Hz, dense wave 50 Hz) and voltage (2–4 V). Electroacupuncture was performed continuously for 4 weeks. | All procedures involving animals were approved by the Animal Protection and Ethics Committee of Xiamen University (XMULAC20190142) and were performed by following the Guiding Opinions on the Good Treatment of Laboratory Animals. | c-myc,Bcl-2,p53 | W. Huang, Y. Yau, J. Zhu et al., 2022 |
| MCAO rats  MCAO control (n=20); EA (n=20) | EA | Acupuncture needles (0.3 mm in diameter) were inserted at a depth of 2 to 3 mm into the skin at the DU20 and DU24 acupoints. The stimulation parameters were set as follows: Waves of 1 and 20 Hz, and 1–3 mA were delivered for 30 min once per day. | All animal procedures were conducted in accordance with international ethical guidelines and the National Institutes of Health Guide for the Care and Use of Laboratory Animals, and all experiments were approved by the Institutional Animal Care and Use Committee of Fujian University of Traditional Chinese Medicine (Fuzhou, China). | MMP‑2,MMP‑9 | R. Lin, K. Yu, X. Li et al.,2016 |
| APP/PS1 rats | EA | The needles were then connected to an acupuncture point nerve stimulator, and stimulation with a frequency of 1/50 Hz and an intensity of 1.3 mA was administered for 15 min. | All animal procedures were conducted in accordance with international ethical guidelines and the National Institutes of Health Guide for the Care and Use of Laboratory Animals | Aβ1-42,LRP1 | WangXin,2017 |
| MCAO rats | EA | The needles were then connected to an acupuncture point nerve stimulator, and stimulation with a frequency of 2/100 Hz and an intensity of 2 mA was administered for 40 min. | All procedures in this study were performed in compliance with the National Institutes of Health Guide for Care and Use of Laboratory Animals. | FITC-NGF in BBB | Y. Zhao, X. Mao, H. Wang et al.,2022 |
| MCAO rats | EA | The needles were then stimulated using an acupuncture point nerve stimulator at an intensity of 2 mA and a frequency of 100 Hz. | All procedures in this study were performed in compliance with the National Institutes of Health Guide for Care and Use of Laboratory Animals. | NGF in BBB | J. Zhang, X. Lin, H. Zhou et al.,2018 |
| SD rats | EA | The current intensity was 3 mA, the frequency was 2/100 Hz, and the stimulation lasted for 40 min. Control rats were treated with the same binding for 40 min. | All procedures were carried out in accordance with the National Institutes of Health Guide for Care and Use of Laboratory Animals. | genes | C. Ma, L. Gan, H. Wang et al., 2022 |

Supplementary Table 2 Characteristics of included human studies.

| Sample size | Treatment | Experimental design | Treatment course | Assessment of experimental outcomes | Refs. |
| --- | --- | --- | --- | --- | --- |
| older participants (n-52) | cyclic chelates (Gadoterate meglumine, Dotarem, Guerbet) | / | 19-28 months after either cyclic or linear Gd agent. | T1-weighted magnetic resonance imaging (MRI) | A. Montagne, M.T. Huuskonen, G. Rajagopal et al.,2019 |
| AD patients (n=60） | probiotic milk containing Lactobacillus acidophilus, Lactobacillus casei, Bifidobacterium bifidum, and Lactobacillus fermentum (2 × 109 CFU/g for each) /milk | RCT | once daily for 12 weeks. | MMSE, blood samples(hs-CRP\|、NO、TAC、GSH、MDA、FPG、TG、HDL、LDL、serum insulin) | E. Akbari, Z. Asemi, K.R. Daneshvar et al.,2016 |
| AD patients (n=79） | selenium plus probiotic containing Lactobacillus acidophilus, Bifidobacterium bifidum, and Bifidobacterium longum (2 × 109 CFU/day each) (n = 27)/selenium (n = 26) / placebo (n = 26) | RCT | Daily for 12 weeks | hs-CRP，HOMA-IR，homeostasis，LDL-cholesterol，HDL-cholesterol，GSH，mini-mental state examination score，QUICKI，compared with only selenium and placebo. Additionally, triglycerides | O.R. Tamtaji, R. Heidari-Soureshjani, N. Mirhosseini et al.,2019 |
| older adults with memory complaints (n=121) | *B. Breve* A1 capsules / placebo | RCT | daily for 12 weeks | RBANS，MMSE, blood parameters | Y. Kobayashi, T. Kuhara, M. Oki et al., 2019 |
| MCI patients (n=100） | DW2009/placebo | RCT | daily for 12 weeks | CNT, Serum BDNF, Fecal Microbiota(bifidobacteria, lactobacilli, and clostridia) | Y.H. Hwang, S. Park, J.W. Paik et al.,2019 |
| healthy elders (n=63) | placebo /probiotics containing Bifidobacterium bifidum BGN4 and Bifidobacterium longum BORI | RCT | daily for 12 weeks | cognitive function and mood tests(CERAD-K, SWLS, GDS-K, PANAS), serum BDNF, microbiota(DNA, 16S rRNA) | C.S. Kim, L. Cha, M. Sim et al.,2021 |
| PSCI patients (n=360) | ACU | RCT | daily for 12 weeks | MMSE, MOCA, CDR, ADL, NPI, QOL, Aβ42, MRI | Du Y, L. Zhang, W. Liu et al.,2020 |
| AD patients (n=14) and normal controls (n=14） | ACU | / | 3 minutes | fMRI-ALFF(Before and after acupuncture), MMSE, MoCA, CDR | W. Zheng, Z. Su, X. Liu et al.,2018 |
| SCD patients (n=60) | EA | / | 24 times | clinical efficacy Z-score, MoCA, microbiota(DNA, 16S rRNA, Escherichia–Shigella, Bifidobacterium) | T. Wang, X. Yan, Q. Zhou,2019 |
| IBS-D paitients  (n=40) | ACU | / | 1 time every other day, 3 times a week, 6 weeks of treatment, a total of 18 times. | IBS-SSS score, microbiota, SCFAs | L. Chen, W.L. Xu, L.X. Pei et al.,2021 |
